# Increased risk of keratopathy after psoriasis: A nationwide population-based study

**DOI:** 10.1371/journal.pone.0201285

**Published:** 2018-07-25

**Authors:** Chia-Yi Lee, Hung-Chi Chen, Hui-Wen Lin, Jing-Yang Huang, Teng-Li Lin, Chia-Hsuan Yang, Chao-Bin Yeh, Hung-Yu Lin, Shun-Fa Yang

**Affiliations:** 1 Department of Ophthalmology, Show Chwan Memorial Hospital, Changhua, Taiwan; 2 Department of Ophthalmology, Chang Gung Memorial Hospital, Linkou, Taiwan; 3 Department of Medicine, Chang Gung University College of Medicine, Taoyuan, Taiwan; 4 Center for Tissue Engineering, Chang Gung Memorial Hospital, Linkou, Taiwan; 5 Department of Optometry, Asia University, Taichung, Taiwan; 6 Department of Medical Research, China Medical University Hospital, China Medical University, Taichung, Taiwan; 7 Department of Medical Research, Chung Shan Medical University Hospital, Taichung, Taiwan; 8 Department of Dermatology, Taichung Veterans General Hospital, Taichung, Taiwan; 9 Department of Medicine, Taipei Medical University Hospital, Taipei, Taiwan; 10 Department of Emergency Medicine, School of Medicine, Chung Shan Medical University, Taichung, Taiwan; 11 Department of Emergency Medicine, Chung Shan Medical University Hospital, Taichung, Taiwan; 12 Institute of Medicine, Chung Shan Medical University, Taichung, Taiwan; 13 Department of Optometry, Chung Shan Medical University, Taichung, Taiwan; 14 Department of Optometry, Yuanpei University of Medical Technology, Hsinchu, Taiwan; 15 College of Health, Chung Chou University of Science and Technology, Changhua, Taiwan; University of Naples, ITALY

## Abstract

**Background:**

To evaluate whether the presence of psoriasis increases the risk of keratopathy incidence by using Taiwan’s National Health Insurance Research Database (NHIRD).

**Methodology/Principal findings:**

This retrospective cohort study used data from the NHIRD for the 2009 to 2012 period. A total of 3,648 patients diagnosed with psoriasis were enrolled in the study group while another 14,592 individuals were selected as the control group. The study group was propensity score-matched with a group of controls who had not received a diagnosis of psoriasis. Multivariate Cox regression analysis was performed to estimate the adjusted hazard ratio (aHR) of keratopathy. For the events of keratopathy, 71 patients in the study group and 208 patients in the control group developed keratopathy with a attributable risk of 23.43 per 100,000 person-months (incidence rate ratio = 1.40; P = 0.01) which correlated to the elevated cumulative probability (P = 0.03). The multivariate analysis revealed that the risk of keratopathy was higher in patients who had psoriasis (aHR = 1.31, P = 0.04). In addition, age older than 60 years (aHR = 2.10, P<0.01) and dry eye disease (aHR = 2.79, P<0.01) would also increase the risk of developing keratopathy.

**Conclusions:**

Psoriasis was associated with an increased risk of keratopathy in patients without preexisting prominent corneal disease. Moreover, the risk of incident keratopathy increases with exposure to psoriasis.

## Introduction

Psoriasis, an autoimmune disease presenting with skin desquamation, redness, and pustular formation,[1] is one of the most common chronic inflammatory dermatological diseases which affects approximately 125 million individuals worldwide.[2] Psoriatic arthritis is a manifestation of psoriasis that occurs in approximately 30% of psoriatic patients.[3] Approximately 75% of cases of psoriatic arthritis emerge after psoriasis has manifested,[4] while arthritis occurs prior to skin lesions in 10% of such patients.[4] In addition, psoriasis is related to systemic diseases such as hypocalcemia, pregnancy, streptococcal infections, diabetes, myocardial infarction, stroke, psychosocial stress, impaired social functioning, ocular rosacea, and death due to cardiovascular disease.[1, 2, 4]

Ocular rosacea, conjunctivitis, and uveitis may develop in patients with psoriasis.[5] Atopic dermatitis, an inflammatory skin disorder with a similar pathophysiology to psoriasis,[6] is associated with ocular lesions such as atopic keratoconjunctivitis.[7] Moreover, dry eye disease (DED) is common in patients with psoriasis with poor tear function, as determined by shorter tear break-up times, low Schirmer test results, abnormal fluorescein patterns, and the presence of meibomian gland dysfunction.[8] The association between corneal disease and psoriasis was first proposed in 1936, when keratitis with inflamed conjunctiva was observed in several cases of psoriasis.[9] Subsequently, punctuate epithelial keratitis, deep corneal opacity, sterile corneal infiltrates, and corneal melting have been observed in patients with psoriasis.[10, 11] However, most of the previous related studies were only sporadic case reports or original study with few psoriasis cases.[9–11] To further substantiate the relationship between psoriasis and keratopathy, a larger case series or even nationwide cohort survey is warranted.

The aim of the present study was to evaluate whether the presence of psoriasis increases the risk of the incident keratopathy by using data from Taiwan’s National Health Insurance Research Database (NHIRD). Other comorbidities possibly related to psoriasis were also analyzed in a multivariate model.

## Methods

### Data source

This retrospective population-based cohort study was approved by the National Health Insurance Administration and the Institute Review Board of Chung Shan Medical University (Registration Number: CSMUH CS2-15061). Provided by the Taiwan National Health Research Institutes, the NHIRD contains insurance claims data of more than 99% of Taiwan’s population. The claims data used in this study were from the 2010 Longitudinal Health Insurance Database (LHID 2010), which contains data on 1 million patients randomly sampled from the registry of the NHIRD for the year 2010. The LHID 2010 data were linked from January 1, 2009 to December 31, 2012, and the International Classification of Diseases (ICD-9) was used to identify the diseases. Medications prescribed for the patients, and the demographics, socioeconomic status and living place of patients were also available in the NHIRD.

### Patient selection

Patients were regarded as having psoriasis if their medical records indicated a history of psoriatic arthropathy (ICD-9 code: 696.0) or psoriasis (ICD-9 code: 696.1). Although psoriatic arthritis and psoriasis do not present with identical lesions, dermatologists in Taiwan use the ICD-9 diagnostic code for psoriatic arthritis to describe “psoriasis with arthropathy”; it was therefore included because excluding it would lead to an underestimation of the occurrence of psoriasis. To avoid misdiagnosis of psoriasis by recruiting similar morbidities (eg, parapsoriasis or pityriasis rosacea), only patients who fulfill the following inclusion criteria would be enrolled in the current study: (1) had received the diagnosis of psoriasis from a dermatologist (department code: 11), (2) received laboratory studies including blood test (exam codes: 08011C, 08013C, 08016C, 08026C, 08036B, and 08126B), biochemistry profile (exam codes: 08005C, 09002C, 09013C, 09015C, 09025C, 09026C) and rheumatoid factor (exam code: 12011C) in dermatological department to exclude possible inflammatory or autoimmune diseases other than psoriasis; and (3) received medical treatment with topical calcitriol (medication code: B023403343), topical retinoid (medication code: B023854343) or topical betamethasone (medication codes: A022192321 and A011652321) after the diagnosis of psoriasis. The index date was set as the first date of psoriasis diagnosis.

To better elucidate the association between psoriasis and keratopathy, the following exclusion criteria were applied: (1) having been diagnosed with legal blindness (ICD-9 code: 369.4); (2) having received any type of eyeball removal surgery (ICD-9 codes: 16.5x); (3) having received an ocular tumor diagnosis (ICD-9 codes: 190.0–190.9); (4) having undergone corneal transplantation (ICD-9 codes: 11.6x, V42.5, 996.51); (5) having received a diagnosis of corneal opacity (ICD-9 codes: 371.0x), interstitial and deep corneal infection (ICD-9 codes: 370.5x), corneal neovascularization (ICD-9 codes: 370.6x) or severe DED with cyclosporine emulsion instillation (ICD-9 codes: 370.33, 370.34, 372.53, 375.15 and 710.2 plus the medication code: BC242064CR) before the index date; (6) having received contact lens-related corneal lesion (ICD-9 codes: 371.24 and 371.82) and progressive high myopia (ICD-9 code: 360.21) throughout the study period to standardize the ocular conditions of participants; and (7) having received a psoriasis diagnosis before 2010. In addition, the individuals in the study group were propensity score matched to four non-psoriasis individuals that serve as the control group which the details will be discussed in the statistic section, and psoriasis patients who could not be matched with four non-psoriasis patients were excluded.

### Main outcome measurement

There is no single ICD-9 diagnostic code for keratopathy to be defined as the primary outcome of the current study. Therefore, the diagnosis of keratopathy in the current study consisted of different disease subgroups: (1) corneal ulcer (ICD-9 codes: 370.0x), (2) superficial keratitis (ICD-9 codes: 370.2x, 370.9x), (3) keratoconjunctivitis (ICD-9 codes: 370.3x, 370.4x), (4) interstitial and deep keratitis (ICD-9 codes: 370.5x), (5) corneal neovascularization (ICD-9 codes: 370.6x), (6) corneal opacity (ICD-9 codes: 371.0x) and (7) corneal edema (ICD-9 codes: 371.21–371.23). In practice, ICD-9 codes for “unspecific corneal disorder” and “unspecific corneal edema” are overused for minor corneal lesions; thus, these codes were eliminated to prevent overestimation of the number of clinically significant keratopathy episodes. Only patients received the above diagnostic code by an ophthalmologist (department code: 10) would be regarded as the achievement of outcome.

### Demographic variables and comorbidities

We also considered the effect of demographic conditions (ie, age, gender, and income level) and the following systemic comorbidities in analysis model to erase the potential risk factor for keratopathy: hypertension (ICD-9 codes: 401–405), diabetes mellitus (DM) (ICD-9 codes: 250.x), acute ischemic heart disease (ICD-9 codes: 410–413), congestive heart failure (ICD-9 codes: 398.91, 402.01, 402.11, 402.91, 404.01, 404.03, 404.11, 404.13, 404.91, 404.93, 425.4–425.9, 428.x), peripheral vascular disease (ICD-9 codes: 093.0, 437.3, 440.x, 441.x, 443.1–443.9, 47.1, 557.1, 557.9, V43.4), cerebrovascular disease (ICD-9 codes: 362.34, 430.x–438.x), dementia (ICD-9 codes: 290.x, 294.1, 331.2), chronic pulmonary disease (ICD-9 codes: 416.8, 416.9, 490.x–505.x, 506.4, 508.1, 508.8), rheumatic disease (ICD-9 codes: 446.5, 710.0–710.4, 714.0–714.2, 714.8, 725.x), peptic ulcer disease (ICD-9 codes: 531.x–534.x), liver disease (ICD-9 codes: 070.22, 070.23, 070.32, 070.33, 070.44, 070.54, 070.6, 070.9, 456.0–456.2, 570.x, 571.x, 572.2–572.8, 573.3, 573.4, 573.8, 573.9, V42.7), hemiplegia or paraplegia (ICD-9 codes: 334.1, 342.x, 343.x, 344.0–344.6, 344.9), renal disease (ICD-9 codes: 403.01, 403.11, 403.91, 404.02, 404.03, 404.12, 404.13, 404.92, 404.93, 582.x, 583.0–583.7, 585.x, 586.x, 588.0, V42.0, V45.1, V56.x) and malignancy including lymphoma and leukemia, but excluding malignant neoplasm of the skin (ICD-9 codes: 140.x–172.x, 174.x–195.8, 200.x–208.x, 238.6). About the ocular diseases, cataract and cataract surgery (ICD-9 codes: 366.10–366.19, 366.8, 366.9 and surgery code: 86008C), uveitis (ICD-9 codes: 363.0x, 363.1x, 363.2x 364.0x, 364.1x, 364.2x and 364.3), glaucoma (ICD-9 codes: 365.x), and DED (ICD-9 codes: 370.33, 370.34, 372.53, 375.15 and 710.2) were enrolled in the analysis model. In addition, several medications that may lead to the development of keratopathy include Amiodarone, non-steroid anti-inflammatory drugs (NSAID, included Aspirin, Indometacin, Diclofenac, Naproxen, Sulindac, Ibuprofen, Celecoxib, Mefenamic acid, Ketorolac, and Pimecrolimus), Hydroxychloroquine, anti-neoplastic medications (included Tamoxifen, Cytarabine), biological disease-modifying antirheumatic drugs (bDMARD, included Etanercept, Adalimumab, Golimumab, Ustekinumab, Tocilizumab, Rituximab, Abatacept, Xeljanz) and antibiotics (included Clarithromycin, Ciprofloxacin) were also considered in the statistical analysis if the medications were prescribed 180 days before or after the index date. The insurance codes for these medications were shown in S1 Table. We longitudinally traced the data from the index date until the date of keratopathy diagnosis, withdrawal from the National Health Insurance program, or December 31, 2013.

### Statistical analysis

SAS version 9.4 (SAS Institute Inc, NC, USA) was employed for all the analysis. First, the propensity score matching was used to deal with the potential confounders for each individual with the application of SAS MARCO program, PROC LOGISTIC program and PROC SQL in SAS software. The propensity score is a value that predicted probability of exposure based on multiple factors used commonly in observational study,[12] which can reduce the selection bias more precisely than the age-gender matching. The factors enrolled in the propensity score matching included birth year, gender, income, all diseases in Modified Deyo-Charlson comorbidity index, cataract and cataract surgery, uveitis, glaucoma and DED in the current study for the psoriasis exposure. The paired psoriasis and control individuals were randomly matched when the difference of propensity score, which calculated by logistic regression, was less than 0.01 between one psoriasis exposure individual and four non-psoriasis individuals. After propensity score matching, chi-square test was employed to test for differences in the demographic data (age, gender, and income level) between the study and control groups. Then the incidence rate (absolute risk), attributable risk (difference of risk, the difference between the incidence rate in exposure group and the incidence rate in control group) incidence rate ratio (IRR), and corresponding 95% confidence intervals (CI) were calculated by Poisson regression. Cox proportional hazard regression that enrolled all the demographic data, prominent ocular diseases, systemic co-morbidities and related medications mentioned above in the multivariate model were adopted to compute adjusted hazard ratios (aHR). We plotted Kaplan–Meier curves to indicate the cumulative incidence proportion of keratopathy between the study and control groups with an interval of four years after the psoriasis diagnosis, and used the log rank test to determine the significant difference between the survival curves. Because most patients in the NHIRD are Han Taiwanese, race was not considered as a covariate. Results with P<0.05 were regarded as statistically significant and a P value of less than 0.01 were depicted as P<0.01.

## Results

**Fig 1:**
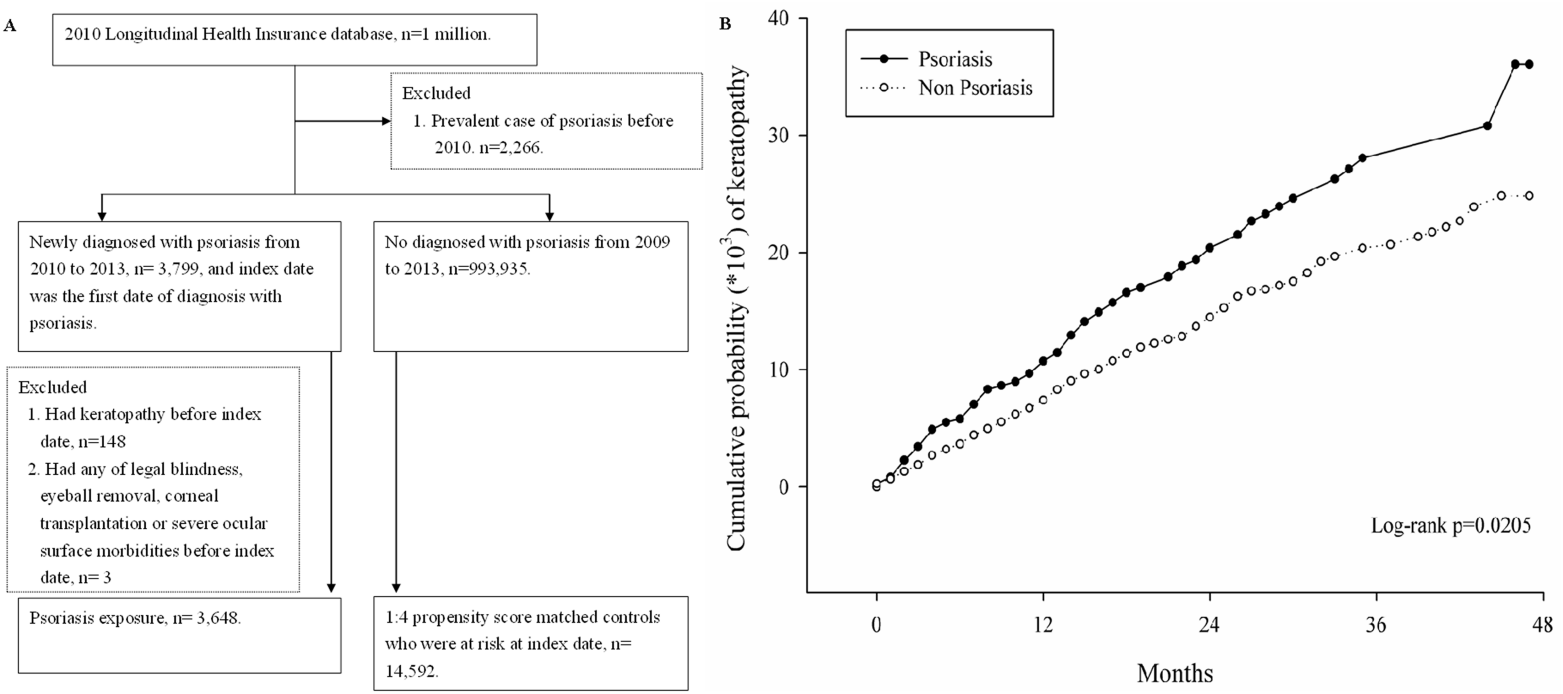
The flowchart of the enrollment of patients with and without psoriasis in the cohort study.

A total of 3,648 patients diagnosed with psoriasis were enrolled in the study group and another 14,592 individuals were selected as the controls (Fig 1). The demographic characteristics were analyzed and the age distribution, gender ratios, income levels, existence of ocular co-morbidities and development of systemic diseases were all similar between the two groups (Table 1). About the keratopathy-related medications, there were higher prescription rates of NSAID and hydroxychloroquine in the study group (Table 2).

**Table 1:** Characteristics in study groups at baseline among psoriasis and propensity score matched control group.

	Psoriasis n = 3,648	Control n = 14,592	P value
Age at index date, Mean ± SD	38.52±20.06	38.64±20.23	0.74
<20	722(19.79%)	2,880(19.74%)	
20–39	1,310(35.91%)	5,224(35.80%)	
40–59	1,010(27.69%)	4,062(27.84%)	
>=60	606(16.61%)	2,426(16.63%)	
Gender			0.98
Female	1,671(45.81%)	6,687(45.83%)	
Male	1,977(54.19%)	7,905(54.17%)	
Low income	49(1.34%)	175(1.20%)	0.48
Ocular co-morbidities			
Cataract and cataract surgery	30(0.82%)	123(0.84%)	0.90
Uveitis	19(0.52%)	71(0.49%)	0.79
Glaucoma	68(1.86%)	257(1.76%)	0.67
Dry eye diseases	178(4.88%)	697(4.78%)	0.80
Modified Deyo-Charlson comorbidity index			0.96
0	2,436(66.78%)	9,732(66.69%)	
1–2	906(24.84%)	3,610(24.74%)	
3–4	207(5.67%)	829(5.68%)	
>=5	99(2.71%)	421(2.89%)	

SD = standard deviation

**Table 2:** The distribution of keratopathy-related medications between the study group and the control group.

	Psoriasis n = 3,648	Control n = 14,592	P value
Amiodarone	11(0.30%)	41(0.28%)	0.84
NSAIDs	1300(35.64%)	4438(30.41%)	<0.01
Hydroxychloroquine	31(0.85%)	36(0.25%)	<0.01
Anti-neoplastic	3(0.08%)	12(0.08%)	1.00
bDMARD	1(0.03%)	6(0.04%)	0.71
Antibiotics	24(0.66%)	85(0.58%)	0.60

NSAID: non-steroid anti-inflammatory drugs

bDMARD: biological disease-modifying antirheumatic drugs

**Fig 2:**
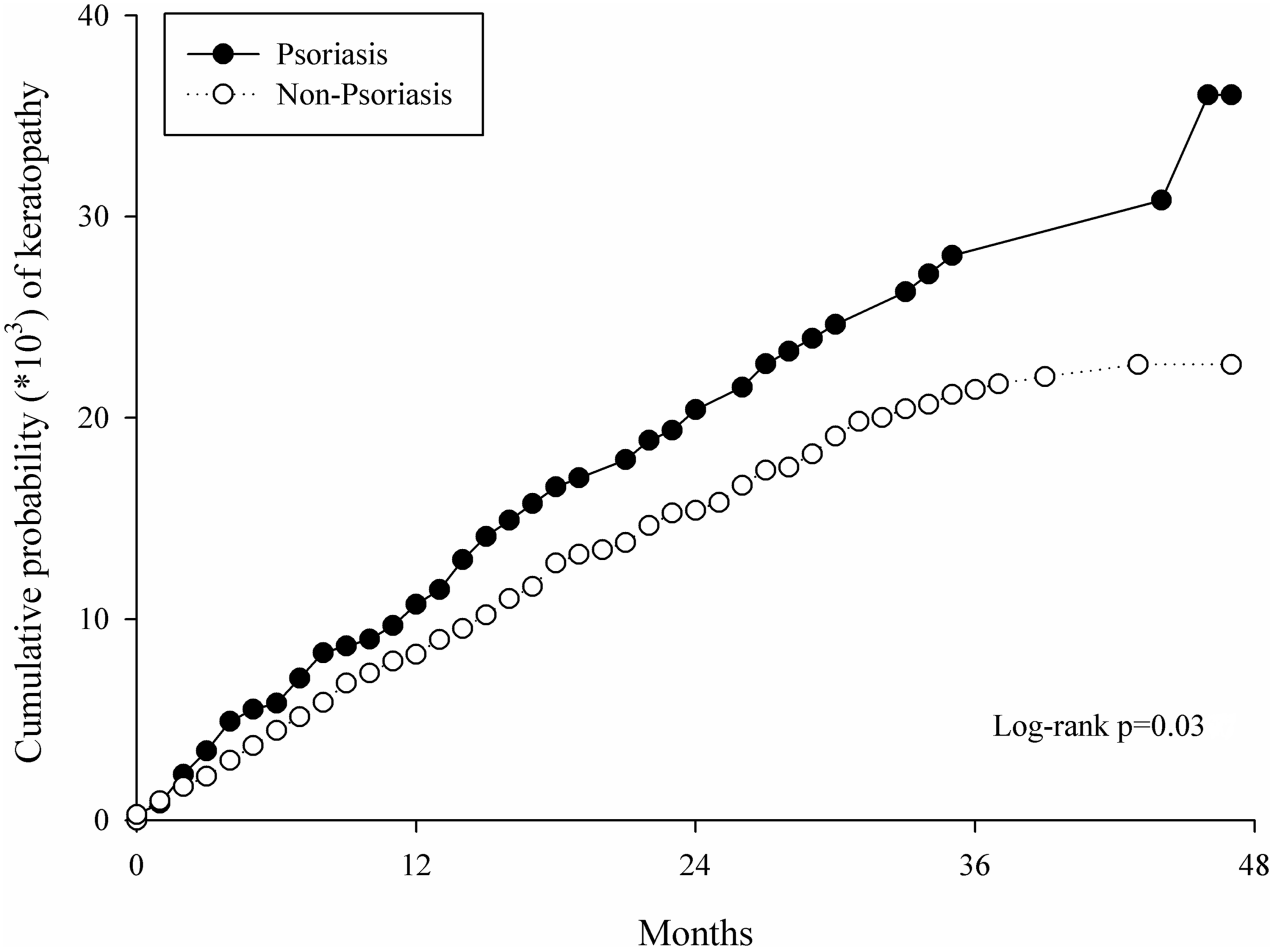
Kaplan-Meier curves with cumulative proportion of keratopathy in the psoriasis and non- psoriasis groups.

For the events of keratopathy, 71 patients in the study group and 208 controls developed different types of keratopathy, with an attributable risk (difference of risk) of 23.43 per 100,000 person-months exposure of psoriasis (Table 3) and IRR of 1.40 (P = 0.01, Table 4). The Kaplan–Meier curves also revealed a higher cumulative probability of keratopathy in the study group (P = 0.03, Fig 2).

**Table 3:** Type of keratopathy with diagnostic code among study groups.

	Psoriasis group (n = 71)	Control group (n = 208)*
Corneal ulcer (370.0)	8(11.27%)	14(6.64%)
Superficial keratitis (370.2, 370.9)	48(67.61%)	132(63.46%)
Keratoconjunctivitis (370.3, 370.4)	13(18.31%)	47(22.60%)
Interstitial and deep keratitis (370.5)	1(1.41%)	2(0.95%)
Corneal neovascularization (370.6)	0(0.00%)	1(0.47%)
Corneal opacity (371.0)	0(0.00%)	8(3.79%)
Corneal edema (371.21–371.23)	3(4.23%)	7(3.32%)

*Since a single patient may diagnosed with more than one keratopathy, the total percentage was not illustrated to avoid confusion

**Table 4:** Incidence of keratopathy between the study group and the control group.

	Psoriasis n = 3,648	Control n = 14,592
Follow up person-months	86,600	355,200
Event of keratopathy	71	208
Incidence rate*	81.99	58.56
Attributable risk *, P value	23.43, 0.01	Reference
IRR (95% CI), P value	1.40(1.07–1.83), 0.01	Reference

* per 10^5^ person-months

IRR, Incidence rate ratio

CI = confidence interval

The Cox multivariate regression analysis revealed a higher risk of keratopathy in patient with psoriasis (aHR = 1.31, P = 0.04). Other potential risk factors for keratopathy included being older than 60 years (aHR = 2.10, P<0.01) and DED (aHR = 2.79, P<0.01), while male gender is a protective factor for keratopathy (aHR = 0.70, P<0.01) (Table 5). Regarding the subgroup analysis, neither gender nor age elevated the risks of developing keratopathy with exposure of psoriasis.

**Table 5:** Hazard ratios of keratopathy and potential risk factors by multiple Cox regression.

	aHR	95% CI	P value
Psoriasis exposure (reference: No)	1.31	1.00–1.72^#^	0.04
Age at baseline (reference: 20–39)			
<20	1.06	0.74–1.52	0.76
40–59	1.04	0.75–1.44	0.82
>=60	2.10	1.48–2.97	<0.01
Gender(reference: Female)			
Male	0.70	0.56–0.89	<0.01
Low income (reference: No)	1.52	0.62–3.69	0.36
Co-morbidities (reference: No)			
Cataract and cataract surgery	1.13	0.46–2.79	0.79
Uveitis	1.51	0.47–4.84	0.49
Glaucoma	1.38	0.77–2.47	0.28
Dry eye diseases	2.79	1.97–3.95	<0.01
Modified Deyo-Charlson comorbidity index (reference:0)			
1–2	1.13	0.85–1.49	0.40
3–4	0.83	0.49–1.39	0.47
>=5	1.10	0.59–2.07	0.76
Medication*			
NSAIDs	1.14	0.89–1.45	0.31
Hydroxychloroquine	0.57	0.08–4.09	0.58
Antibiotics	0.52	0.07–3.74	0.52

aHR = adjusted hazard ratio

CI = confidence interval

NSAID: non-steroid anti-inflammatory drugs

bDMARD: biological disease-modifying antirheumatic drugs

^#^ The confidence interval of psoriasis exposure is 1.0046–1.7238 if calculated to forth decimal point

* Amiodarone, anti-neoplastic medications and bDMARD were excluded form multivariate regression analysis after model selection

## Discussion

Psoriasis is associated with the development of various keratopathy events according to the multivariate model in the current study. The multivariate Cox regression analysis results also reveal age older than 60 and DED as risk factors for keratopathy. These findings are consistent with those of previous research[9–11] and demonstrate that keratopathy may develop after psoriasis.

Psoriasis is a common dermatological disease with prevalence up to 2.9% throughout the world,[13] while Taiwan’s prevalence is nearly 0.3% which reaches the average of non-Caucasian population.[13] According to recent research, DED is associated with elevated IL and TNF levels in tear film and various systemic inflammatory diseases, including psoriasis.[14, 15] The immune imbalance and tear film instability in concurrent psoriasis and DED may cause decreased corneal sensitivity and ocular surface damage,[16–18] which may increase the likelihood of keratopathy. Furthermore, the corneal hysteresis and corneal resistance factor are decreased in patients with psoriasis,[19] suggesting that the corneal structure is weakened in patients with this condition. Collectively, the aforementioned evidence suggests a relationship between psoriasis and keratopathy,[14–19] which correlates with the results in the present study.

Previous reports have revealed cases with concurrent psoriasis and keratopathy.[10, 11, 20] For example, Kilic *et al* reported on a total of 100 patients with psoriasis, 16 of whom were also diagnosed with keratopathy, including punctate epithelial erosion, vascularization, corneal opacity, and filament keratopathy.[10] The results of our study are compatible with those of Kilic *et al* except that our incidence rate is lower. However, the mean duration of psoriasis in that study was nearly 10 years,[10] whereas the longest disease interval in the present study was four years. According to our results, a longer disease interval of psoriasis is associated with a higher likelihood of developing keratopathy, as indicated by the cumulative probability in the study group (P = 0.03). Moreover, the time-sequence in the study by Kilic *et al* was not evaluated,[10] and patients with keratopathy before the emergence of psoriasis might have been included in the study group.

Psoriasis could be an independent risk factor of keratopathy in patients without prior major corneal disease after multivariate adjustment with several risk factors. In addition, DED also increased the risk of developing keratopathy with aHR of 2.79 (P<0.01), which is a risk factor for keratopathy according to previous study.[18] Moreover, NSAID would increase the risk of keratopathy in previous experience,[21] but in the current study the NSAID only marginally elevated the risk of keratopathy which may because certain mild-degree keratopathies were excluded.

Psoriasis is related to numerous systemic diseases, [22, 23] but the multivariate analysis results show no association between any systemic diseases. Notably, DM is associated with several ocular surface disorder,[24] and we speculate that metabolic diseases with a disease interval less than 5 years are unlikely to induce keratopathy or even retinopathy.[25] Moreover, patients older than 60 years tended to have keratopathy which may because aging is related to DED and ocular surface infection.[26]

There are several limitations in the current study. First, the retrospective nature restricted the precision of our study, although we matched the study group by propensity score with the controls. Second, some self-paid managements like refractive surgery or contact lens purchase are not available in this national-insurance database and we can only exclude relevant diseases to standardize the ocular condition in the two groups. Third, the specific relationships between psoriasis and different types of keratopathy, such as epithelial defects or stromal opacity, were not evaluated separately, although various forms of keratopathy have been reported in patients with psoriasis.[10, 11, 20]

In conclusion, psoriasis is associated with the development of keratopathy in patients without previously prominent corneal disease (attributable risk: 23.43 per 100,000 person-months after psoriasis exposure). Furthermore, the risk of incident keratopathy is increased in individuals with longer psoriasis duration. Further large-scale prospective research is necessary to validate whether the degree of psoriasis and extent of keratopathy are related.

## Supporting information

S1 Table List and code of keratopathy-related medication. (DOCX)
