# Supplementary material for: Increased risk of keratopathy after psoriasis: A nationwide population-based study
Source: PLoS One. 2018 Jul 25;13(7):e0201285. doi: 10.1371/journal.pone.0201285 (PMC6059472; doi:10.1371/journal.pone.0201285)
Supplement: S1 Table — (DOCX) [file pone.0201285.s001.docx]

**Supplementary Table 1: List and code of keratopathy-related medication**

**Cardiovascular agents:**

Amiodarone: A046077100, B014861216

**NSAID:**

Mefenamic acid: A008774100, A042129100, A005249100, A036962100

Ketorolac: AC46348209, AC45965100, A047784209

Diclofenac: B020157100, BC201571G0, A035037329, A035037335, A0453581G0

Sulindac: AC30612100, A031020100

Celecoxib: BC23177100

Aspirin: A023534100, A024465100, A036599100, A0429341G0, A044016100, A0440161G0, AC37344100, AC373441G0, AC37702100, AC41220100, AC41814100, AC418141G0, AC42774100, AC42934100, AC43309100, AC433091G0, AC43664100, AC436641G0, AC44176100, AC441761G0, AC495361G0, BC24025100, BC240251G0

Ibuprofen: AC34804100, AC45438151, AC30466100, AC28321100, AC43152151

Naproxan: AC153871G0, AC43823100, A026707100, A029222500

Indomethacin: NC08167100, AC41296100, AC011541G0

Pimecrolimus: BC23645335

**Antimalarial:**

Hydroxychloroquine: BC22376100, B022376100, AC50142100

**Anti-neoplastic:**

Tamoxifen: BC22154100, B022154100

Cytarabine: BC21695209, BC21695221, BC21695238

**bDMARD:**

Etanercept: KC00851240

Adalimumab: KC00776283

Golimumab: KC00911206

Ustekinumab: KC00957206

Tocilizumab: KC00907219

Rituximab: KC00928248

Abatacept: KC00897265

Xeljanz: BC26219100

**Antibiotics:**

Clarithromycin: AC45343100, AC46638100, A049305100, AC57840100

Ciprofloxacin: BB17692100, BB21062100, AC3808910, AC45201100, AC37688245
